# Insights from the molecular docking and simulation analysis of P38 MAPK phytochemical inhibitor complexes

**DOI:** 10.6026/97320630019323

**Published:** 2023-03-31

**Authors:** Chennu MM Prasada Rao, Kotaiah Silakabattini, Naidu Narapusetty, V. Jhansi Priya Marabathuni, Karavadi Thejomoorthy, Tanniru Rajeswari, Sabitha Y

**Affiliations:** 1 Department of Pharmaceutical Chemistry, Raffles University, Neemrana, Rajasthan - 301705; 2 Department of Pharmacy, Komar University of Science and Technology, Iraq; 3 Bellamkonda institute of Technology and sciences, Podili, A.P - 523240; 4 Bellamkonda institute of Technology and sciences, Podili-A.P-523240; 5 Malineni Lakshmaiah College of Pharmacy, Singarayakonda, A.P - 523101; 6 Department of Pharmaceutical Chemistry, Raffles University, Neemrana, Rajasthan-301705; 7 Ciencia life sciences, Sardar Patel Nagar, Nizampet, Hyderabad, Telangana State, India

**Keywords:** p38MAP kinases, molecular dynamic simulations, molecular docking, phytochemicals

## Abstract

It is of interest to develop p38α MAPK inhibitors. Docking, ADMET properties calculation, molecular dynamics, and MM-PBSA approaches were used to investigate the therapeutic potentials of p38α MAPK in complex with SB203580 (1A9U). The photo-molecules metergoline, withaphysacarpin, philadelphicalactone, canthin-6-one 9-glucoside, and SB-21600011 demonstrated high binding affinity compared to the reference drug. Furthermore, ADME profiles validated the drug-like properties of the prioritized phyto-compounds. Besides that, MD simulations were performed along with reference inhibitors for withaphysacarpin and metergoline to assess stability. Binding free energy calculations (MM-PBSA) revealed that metergoline and withaphysacarpin had estimated values (G) of 97.151 ± 21.023 kJ/mol and -82.084 ± 15.766 kJ/mol, respectively. In this study, metergoline and withaphysacarpin were found to have high affinity against p38α MAPK when compared to the reference compound SB 203580.

## Background:

The first p38 MAPK isoform identified was p387alpha;, a 38-kDa protein that rapidly phosphorylated tyrosine in response to LPS stimulation. The cloned p38 cDNA binds pyridinyl imidazole derivatives and suppresses inflammatory cytokines including IL-1 and TNF in LPS-stimulated monocytes. UV radiation, heat, osmotic shock, and oxidative stress from cytokines, chemokines, hormones, and growth activate p38α. P38α signaling helps cells adapt to environmental changes [1]. Docking and MD simulations were used to study MAPK-phytochemical interactions. It can even execute virtual screening tests on a huge number of compounds, score the results, and provide a structural theory of how the ligands blocked the target, which helps lead optimization. Docking studies predicted preferred orientation, affinity, and interaction, which helped predict binding affinity and understand molecular mechanisms [2].

## Materials and Methods:

## Preparation of Protein/Receptor:

The crystalline structure of MAP KINASE P38 complex along with an inhibitor SB203580 (PDB ID- 1A9U) with resolution 2.5 Å was downloaded from Research Collaboratory for Structural Bioinformatics (RCSB) which is the protein data bank database (www.rcsb.org), in .pdf format. The 1A9U protein contains a single chain (Wang et al. 1998). Ahead of the detailed docking analysis, 'A' chain was selected as the target for this study, and then the protein was prepared using AutoDock tool [3]. The selected protein complex had an inbuilt ligand 4-[5-(4-Fluoro-Phenyl)-2-(4-Methanesulfinyl-Phenyl)-3h-Imidazol-4-Yl]-Pyridine. Both the water molecules and non-interacting ions, along with the native ligand eliminated from the crystal structure. The missing hydrogens were added in order to alleviate the stress of the crystal structure and make the protein accessible for use in the AutoDock docking simulation program. After the structural reduction, the protein was prepared using AutoDock Tools (version1.5.6) (ADT), the graphical user interface, which concerns the addition of hydrogen atoms, Gasteiger charge calculations, and merging of the non-polar hydrogens to carbon atoms. The generated macromolecular structure was saved as a pdbqt file.

## Ligand Preparation:

Most of the ligands were collected from PubChem database (https://pubchem.ncbi.nlm.nih.gov/) [4]. The ligands were downloaded in .sdf format and then converted into .mol files using the chimera software. Ligand input files for docking prepared using AutoDock Tools and saved as pdbqt files.

## SwissADME:

Swiss ADME is a free tool available on the web to grade not only the pharmacokinetics and drug-likeness but also the friendliness of the medicinal chemistry of small molecules. This tool provides free availability to a group of high-speed yet very accurate predictive models for physicochemical properties and pharmacokinetics including in-house adept methods like the BOILED-Egg, iLOGP, and Bioavailability radar [5].

## CHIMERA:

UCSF chimaera visualized and analyzed interactions. This extensible application analyses molecular structures and related data including density maps, sequence alignment findings, docking results, trajectories, etc. It offers basic services, visualization, and extensions with high functionality. This framework ensures the extension operation meets developer needs for additional functionality.

## Discovery Studio:

The Discovery Studio helps in the identification of interactions between the active sites in the target and ligand conformation, along with the type of interaction and bond distances. Discovery studio is one unique, centralized and easy to use, graphical interface used for powerful drug designing and modelling of protein (Discovery Studio, 2008).

## Docking:

The molecular docking method was applied to the selected ligands with the help of AutoDock Vina [6, ,8,9], an automated molecular docking and virtual screening software. A grid box with the dimensions X: 30, Y:30, Z:30 Å and a grid spacing of 1.0 Å focused at X: -5.61, Y:17.289, Z: 26.212 was identified as the protein target docking site. Using AutoDock Vina as a secondary docking program, the best molecular interacting inhibitors were observed. The interactions between the active sites in the target and ligand conformation, along with the type of interaction and bond distances, were identified using Discovery Studio Visualizer.

## Molecular dynamic simulation:

In the current investigation, we employed the MD simulations for the reference and two selected compounds of targeted protein p38 MAPK using Groningen Machine for Chemical Simulation (GROMOS) 54A7 force-feld39 using GROMACS suit (version 2019.4) [10,12]. To understand the dynamic behaviour, mode of binding and inhibitor specificity for all the systems, ran simulations 25 ns. Automated Topology Builder (ATB)41 was used for the generation of force-field parameters. The initial structure was solvated using the extended simple point charge (SPC/E) water model. All the systems were immersed in a cubic box of SPC/E water molecules with a minimum distance of 10 Å between the protein surface and the edge of the box. The solvated system is neutralized by adding the counter ions. Energy minimization performed for releasing the conflicting contacts, using the steepest descent method with a tolerance of 10 kJ mol−1. Energy-minimized systems were subjected to equilibration phase-I in which all the heavy atoms were position restrained for 2 ns in the NVT ensemble. Further, this is followed by the secondary phase in the NPT ensemble for 2 ns. All the systems were kept at a constant 300 K in association with the velocity-rescale thermostat with a coupling constant of 0.1 ps. All the bonds length is constrained using the LINCS algorithm and SETTLE algorithm used to constrain the geometry of water molecules. The trajectories of MD simulations are analyzed by built-in modules of Gromacs with in house scripts. Root Mean Square Deviation (RMSD), Radius of Gyration (Rg), Root Mean Square Fluctuation (RMSF), Solvent Accessible Surface Area (SASA), Hydrogen bond interactions and stability of various non-covalent interactions analyzed [13].

## Results and Discussion:

Several solved p38α MAPK structures can be found in the Protein data bank (PDB) database [14, 15]. The PDB ID: 1A9U protein structure was solved using x-ray diffraction at 2.5 resolution and is bound to the inhibitor SB203580. The structure is a monomer with 379 residues and two domains that include N- and C-terminal domains. Based on the literature, 1172 phytochemicals were chosen for this study. The SwissADME tool generated the pharmacokinetic and drug-likeness ADMET (Chemical absorption, distribution, metabolism, excretion, and toxicity) parameters of these 1172 compounds. After evaluating the ADME parameters, the Lipinski Rule of Five was used to filter and choose 514 compounds for the docking investigation. The top twelve chemicals were compiled using the information from the SwissADME analysis (Table 1). Using AutoDock Vina, 514 molecules were docked individually with the MAPK Crystal structure (1A9U) [16].

## Pharmacological Profiling of Hit Compounds:

**Figure 1:**
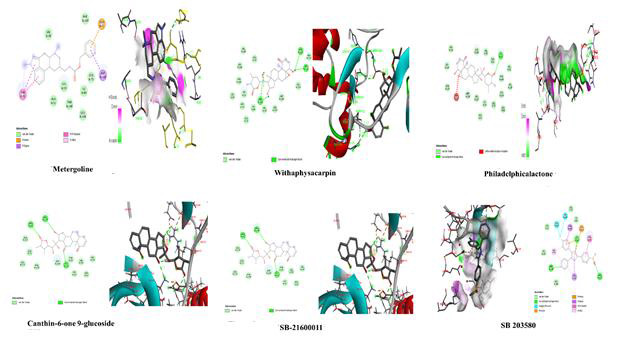
Molecular docking analysis of p36 MAPK inhibitors

The Pharmacokinetic properties (ADMET) are of important aspects in the drug development pipeline. To evaluate drug ability of compounds Lipinski’s rule of five, physically important descriptors and pharmaceutically relevant ADMET properties were evaluated using the Swiss ADME module. The GI absorption measures absorption of orally administered drugs, all the top compounds were predicted high absorption. Additionally, 10 out of the 12 compounds were predicted as P-gp substrates with likely decrease in drug bioavailability except Canthin-6-one 9-glucoside and SN00005348. CYP3A4 is responsible for the metabolism of about 50% of all drugs, except Canthin-6-one 9-glucoside other four top compounds were not CYP3A4 inhibitors. All compounds following Lipinski's rule of five for evaluating drug-likeness. ADME properties are presented in Table 1. Molecular Docking results of five selected compounds tabulated in Table 2 and Figure 1.

Metergoline has the lowest docking score of -9.5 K Cal/mol but no H-bonds, while Withaphysacarpin has the second lowest at -9.4 K Cal/mol but four H-bonds. As a consequence, Withaphysacarpin is more stable than the other compounds in this investigation and can bind the target p38 MAPK amino acid residues and inhibit ATP binding and hydrolysis. Following virtual screening, phytochemicals were selected for MD simulation to study structural changes following contact. At 300K, 1bar, and 25 ns, Gromacs 4.6.2 simulated all complexes. Withaphysacarpin, Metergoline, and SB 203580 were simulated in MD. RMSD, RMSFs, RG, SASA, H-bond analysis, and conformational changes throughout time were evaluated.

## Root Mean Square Deviation of the Complexes:

**Figure 2:**
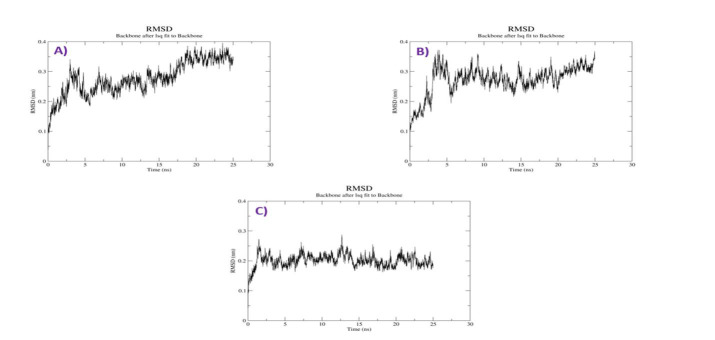
RMSD analysis. (A) Ligand RMSD plot of p38MAPK and Withaphysacarpin (B) p38MAPK and Metergoline (C) p38MAPK and Reference compound (SB 203580)

RMSD values during the simulations were noted. In general, RMSD infers the magnitude of a group of atoms' divergence from the respective original reference structure (protein, ligand, or even ligand-protein complex) [17]. As a result, large RMSD values would be linked to severe instability, linked to changes in the examined molecule's conformation. The plots of the RMSD value against the simulation time are shown in Figure 2A, B, C. RMSD analysis reported a stable value of 0.35 nm at 5 ns and was retained up to 25ns for Withaphysacarpin docked complex. The sharp increase in backbone deviation (maximum of 3.5 A) of this complex found up to 2.5ns course of simulation and then started converging after 5ns with minor variations. Protein-Withanolide Complex shows lower deviation initially and obtained equilibrium after 5 ns and maintained till the end of the simulation, and an average it maintains 0.3 nm throughout the simulation. Protein and Metergoline complex RMSD value held an average RMSD of 0.25 nm till about 18 ns, where a sharp rise to an average of 0.35nm was observed till the end of the 25 ns simulation period. Protein and SB203580 complex have stabilized at 4 ns with RMSD value average 0.2 nM till 25 ns. Protein- Withaphysacarpin Complex showed lower deviation in comparison to protein Metergoline complex.

## Root Mean-Square Fluctuation of the Complexes:

**Figure 3:**
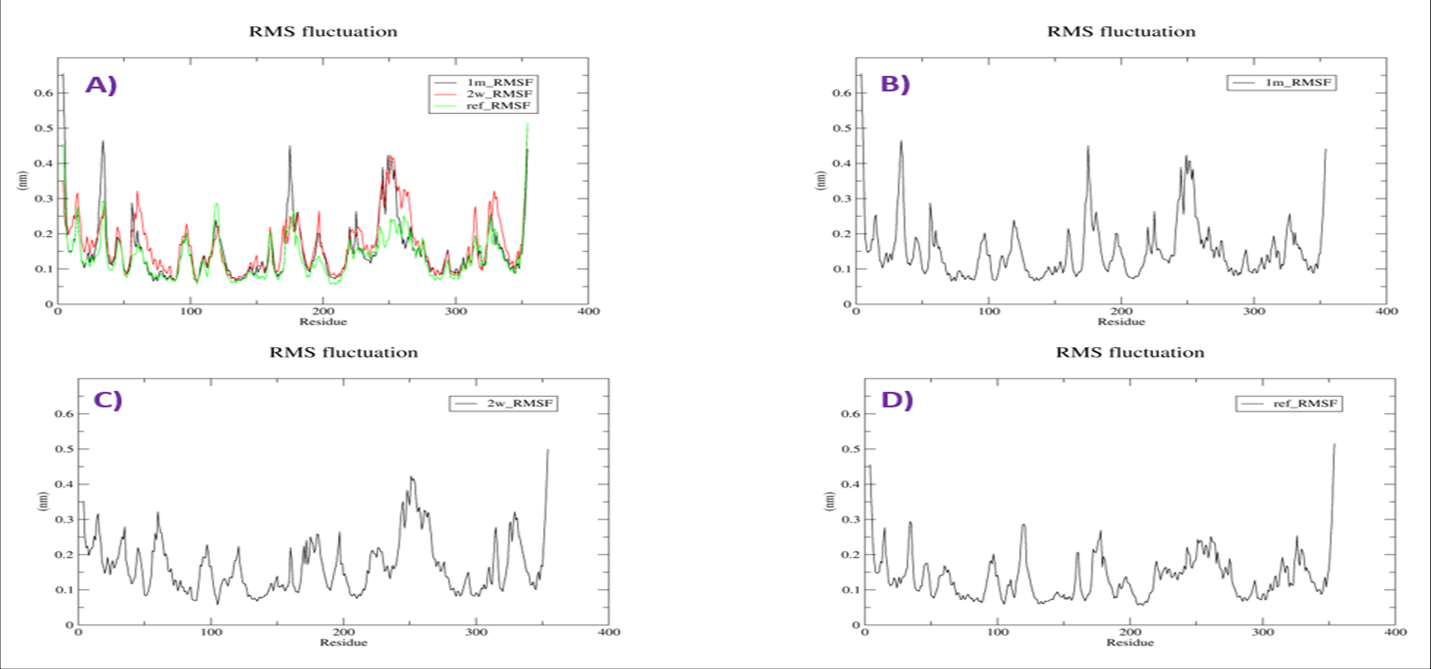
(A) Calculated RMSF plot of docking complexes [p38MAPK - Withaphysacarpin (1m) (Blue), p38MAPK - Metergoline (2w) (Red), and p38MAPK - reference compound (SB 203580) (Green)] (B) RMSF plot [p38MAPK - Withaphysacarpin (1m)] (C) RMSF plot [p38MAPK - Metergoline (2w)] (D) RMSF plot [p38MAPK - Reference compound (SB 203580)].

For gaining more insights regarding the stability of the complex-binding site, per residue versus root-mean-square fluctuation (ΔRMSF) profile estimated for each ligand-bound protein (Benson and Daggett, 2012). Regarding the flexibility of the catalytic residues, almost all amino acids depicted significant ΔRMSF values below 0.3 Å with respect to their Cα atoms, inferring the high flexibility indices for these residues. RMSF value of protein-Withaphysacarpin complex fluctuates from a range of 0.15-0.40 nm in the entire simulation period. When compared with SB203580, Withaphysacarpin fluctuating same pattern except at 240 to 260 amino acids range. RMSF value of 3D87- Metergoline complex fluctuates from a range of 0.2-0.45 nm in the entire simulation period; Fluctuations observed at 30 to 40, 170 to 185 and 240 to 260 residues were high. These two compounds showing similar rmsf compared to SB203580 in catalytic site (Figure 3).

## Radius of Gyration of the Complexes:

**Figure 4:**
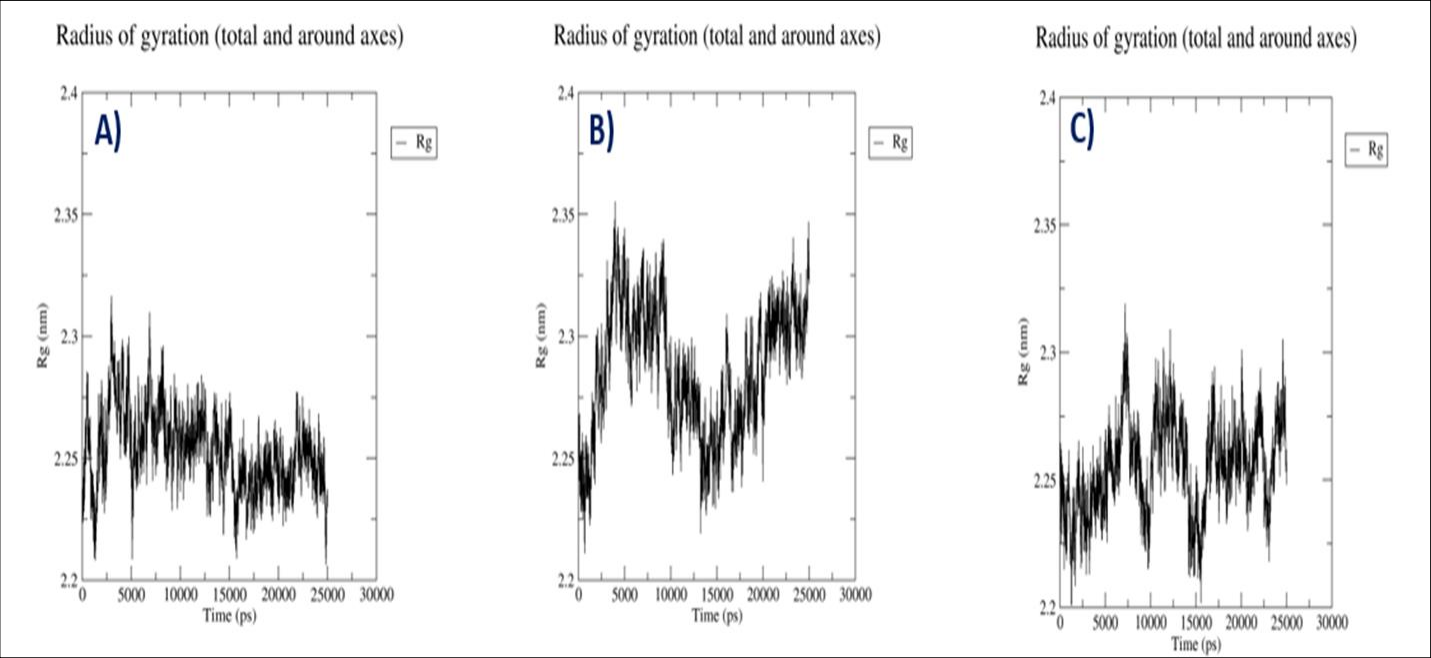
RG analysis. (A) Predicted Rg plot of docking complex [p38MAPK - Withaphysacarpin] (B) Predicted Rg plot of docking complex [p38MAPK - Metergoline] (C) [p38MAPK - Reference compound (SB 203580)].

The radius of gyration (Rg) determines root mean square distance of an object's pieces from its centre of gravity or a given axis. Protein-Withaphysacarpin complex Rg values are not steady throughout 25 ns simulation time and reached maxima at 5ns with 2.35nm again it falls at 15 ns and reached maxima at 25 ns. Protein- Metergoline complex Rg values steady throughout 25ns simulation time indicates interactions between ligand and protein to be stronger. Compared to the reference compound Metergoline shows low Rg values (Figure 4).

## Solvent-accessible-surface area of the complexes:

**Figure 5:**
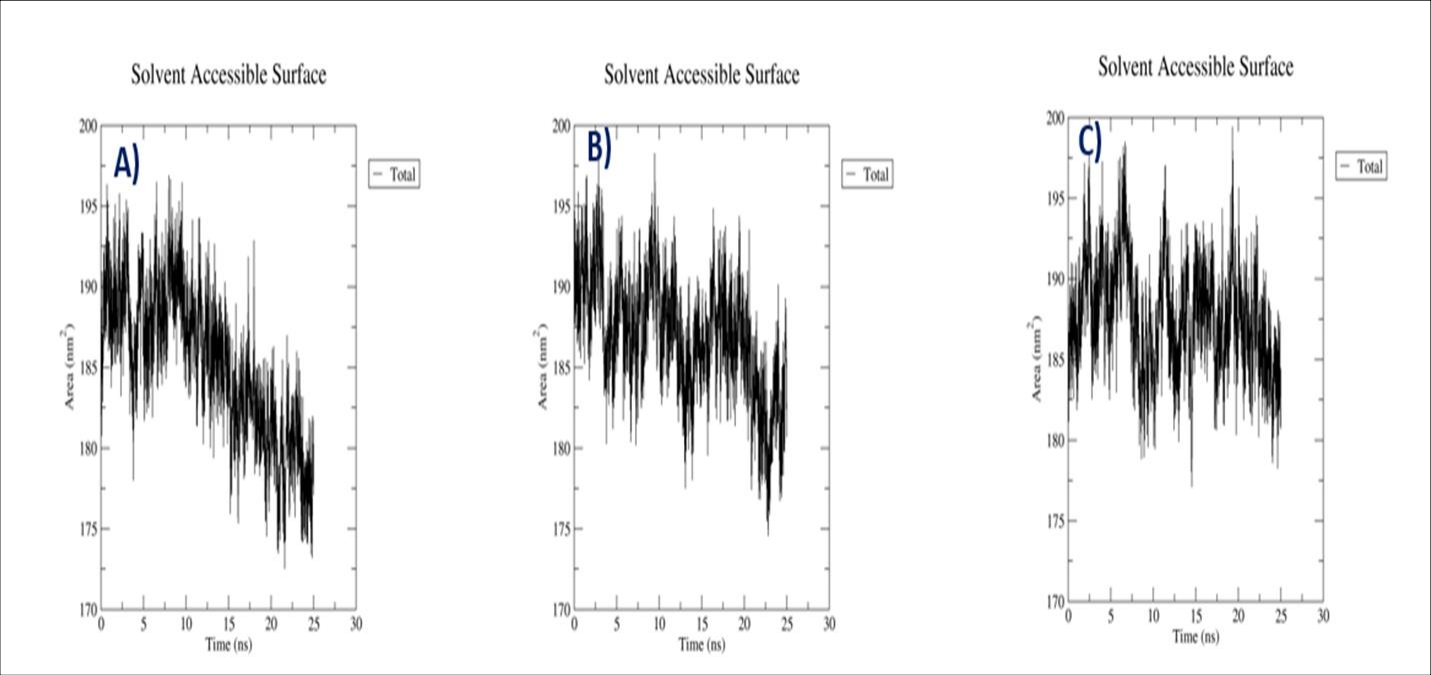
SASA Analysis. (A) SASA plot of selected complex [p38MAPK - Withaphysacarpin] (B) [p38MAPK - Metergoline] (C) [p38MAPK - Reference compound (SB 203580)].

Solvent accessible surface area (SASA) measures the surface area accessible to a solvent. SASA of the ligand-binding site plays an essential role in protein-ligand binding affinity. SASA for this study computed with respect to time and observed that Protein- Metergoline complex exhibited SASA value between 195 nm2 to 180 nm2 until 15ns after that it reached 175 nm2. 3017- Tixocortol complex SASA value 160 nm2. This signified a greater magnitude of flexibility and instability of 3017- Tixocortol complex. Protein-Withaphysacarpin complex exhibited SASA value average 190 nm2 throughout 25 ns (Figure 5).

## H-bond analysis of complexes:

**Figure 6:**
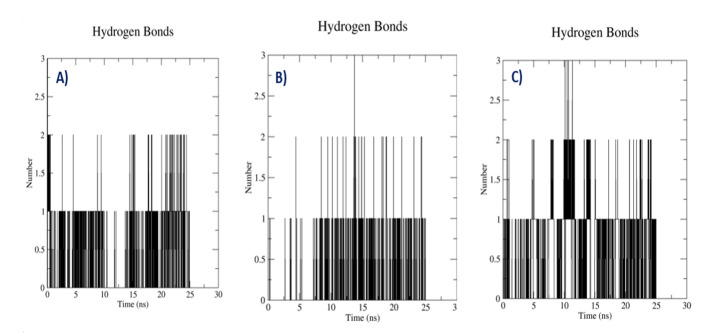
(A) Hydrogen Bond Analysis of docking complex [p38MAPK - Withaphysacarpin] (B) [p38MAPK - Metergoline] (C) [p38MAPK - Reference compound (SB 203580)]

To understand Protein-ligand complex conformational changes and stability required knowledge of hydrogen bond network between protein residues and ligands in MD simulation. During the simulation period, several hydrogen bonds form between protein and ligand and will break. Consequently, observed that protein-Withaphysacarpin complex, 3D8-diosgenin complexes and protein-Metergoline complex forming an average of 2.0 hydrogen bonds. Even SB203580 is showing average 2.0 hydrogen bonds throughout 25ns (Figure 6). The presence of hydrogen bonds is essential for the protein and ligand activity.

## Binding-Free Energy:

The binding free energy decomposition of all the complexes has been summarized in Table 3 and the average binding free energy of protein with three compound complexes were analyzed to be -82.084 ± 15.766 kJ/mol -95.151 ± 21.023 kJ/mol and-97.714 ± 12.725 kJ/mol, respectively. Apart from overall binding free energy, MM/PBSA binding energy of all the drug-target complexes was decomposed to identify the governing factors responsible for stable complex formation. Free energy calculations revealed that vdW energy has a major contribution in binding free energy for all the complexes that make the complex transiently stable. The van der Waal energy and Electrostatic energy contribution for Withaphysacarpin, Metergoline and SB203580 complexes were -127.958 ± 17.132 kJ/m, -142.044 ± 11.024 kJ/mol, -141.072 ± 12.341 kJ/mol. -12.696 ± 6.795 kJ/mol, -54.087 ± 14.773 kJ/mol and -17.026 ± 7.318 kJ/mol respectively. The Metergoline complex significantly favorable change in the vdW energy term was observed. The net binding energy for the Metergoline (-97.151 ± 21.023 kJ/mol) complex revealed stronger system stability compared to the Withaphysacarpin (-82.084 ± 15.766 kJ/mol) and reference compound complexes (-95.714 ± 12.725 kJ/mol).

## Conclusion:

We concluded Metergoline and Withaphysacarpin has the ability to inhibit p38 MAPK according molecular docking study and MD simulations. Based on our findings, we can confirm that Metergoline and Withaphysacarpin can bind and block the active site residues of p38 MAPK. However, more in vitro and in vivo research is required to understand the compound’s inhibitory capacity.

## Figures and Tables

**Table 1:** properties of top twelve candidate ligands

**NAME**	**SN00003509**	**72341**	**637482**	**SN00005348**	**10070979**	**11038269**	**15560508**	**21600010**	**21600011**	**21635715**	**44567005**	**Belotecan**
MW	404.52	492.76	398.37	408.53	424.44	488.61	479.63	472.57	472.57	478.49	488.61	434.51
#Rotatable bonds	6	5	3	6	1	2	7	3	3	8	2	5
#H-bond acceptors	2	3	8	4	7	7	5	7	7	8	7	5
#H-bond donors	2	4	4	1	1	3	1	3	3	3	3	2
TPSA	47.7	76.35	133.75	62.66	99.13	116.59	53.72	121.13	121.13	114.68	116.59	96.34
Lipinski #violations	0	0	0	0	0	0	0	0	0	0	0	0
Ghose #violations	0	4	0	0	0	2	2	0	0	0	2	0
Veber #violations	0	0	0	0	0	0	0	0	0	0	0	0

**Table 2:** Molecular Docking results of five selected compounds

**S. No.**	**Compound**	**Docking Score (KCal/mol**	**Protein and compound interaction**
1	Metergoline (CID: 28693)	-9.5	Val:38, Phe:169, Leu:75, Ile:84, Leu:104, Thr:106, Lys:53, Ala:51-van der waals; Glu:71-Pi-Anion; Asp:168-Pi-Sigma; Tyr:35-Pi-Pi Stacked.
2	Withaphysacarpin (CID: 44567005)	-9.4	Ile:84, Glu:71, Leu:75, Leu:86, Lys:53, Thr:106, Leu:104, Val:105, Ala:51, Ala:111, Gly:110,Met:109, Leu:167, Val:38-van der waals; Asp:168, Asn:115, Asp:112-Conventional Hydrogen Bond.
3	Philadelphicalactone (CID: 11038269)	-9.1	Asn:115, Gly:110, Ala:111, Val:38, Asp:168, Ala:51, Val:52, Thr:106, Val:105, Leu:104, Leu:86, Leu:75, Ile:84, Glu:71, Leu:167, Met:109-Van der waals; Lys:53-Conventional Hydrogen Bond; Asp:112-Unfavorable Acceptor-Acceptor.
4	Canthin-6-one 9-glucoside (CID: 637482)	-8.7	Gly:170, Phe:169, Ala:172, Leu:55, Ile:84, Thr:106-Van der Waals; Lys:53, Arg:67, Glu:71- Conventional Hydrogen Bond; Arg:173-Unfavorable Donor-Donor; Asp:168-Pi-Anion; Val:38-Pi-Sigma; Tyr:35-Pi-Pi-Stacked; Ala:51-Pi-Alkyl.
5	SB-21600011	-8.4	Ala:172, Glu:71, Leu:171, Phe:169, Asp:168, Tyr:35, Gly:31, Val:38, Ala:51, Val:30-Van der Waals; Arg:67, Arg:173, Lys:53-Conventional Hydrogen Bond.
6	Reference Compound (SB 203580)	-9.4	Leu:108, His:107, Leu:86, Leu:75, Ile:84-Van der Waals; Lys:53, Met:109, Arg:173- Conventional Hydrogen Bond; Leu:104, Val:105-Halogen (Fluorine); Asp:168-Pi-Cation; Thr:106, Val:38-Pi-Sigma; Thr:35-Pi-Pi-Stacked; Ala:51-Pi-Alkyl.

**Table 3:** MM-PBSA summary results for protein-ligand complex and values correspond to mean and standard error

	**Withaphysacarpin**	**Metergoline**	**Reference compound**
Van der Waal energy	-127.958 ± 17.132 kJ/mol	-142.044 ± 11.024 kJ/mol	-141.072 ± 12.341 kJ/mol
Electrostatic energy	-12.696 ± 6.795 kJ/mol	-54.087 ± 14.773 kJ/mol	-141.072 ± 12.341 kJ/mol
Polar solvation energy	71.864 ± 16.993 kJ/mol	116.718 ± 18.480 kJ/mol	-141.072 ± 12.341 kJ/mol
SASA energy	-13.295 ± 1.232 kJ/mol	-15.738 ± 1.421 kJ/mol	-141.072 ± 12.341 kJ/mol
Binding energy	-82.084 ± 15.766 kJ/mol	-97.151 ± 21.023 kJ/mol	-141.072 ± 12.341 kJ/mol
